# Heart Failure with Preserved Ejection Fraction (HFpEF), Pulse Wave Velocity, and Heart Rate Recovery Interconnections—A Brief Literature Review

**DOI:** 10.3390/jcm14248781

**Published:** 2025-12-11

**Authors:** Bogdan-Simion Suciu, Vladiana Romina Turi, Simina Crisan, Constantin Tudor Luca, Daniela-Cornelia Lazar, Adelina Andreea Faur-Grigori, Manuela Petrescu, Andreea Dache, Flavius Cioca, Cristina Văcărescu, Dragos Cozma

**Affiliations:** 1 Doctoral School, “Victor Babeș” University of Medicine and Pharmacy, 300041 Timisoara, Romania; bogdan.suciu@umft.ro (B.-S.S.); andreea.faur@umft.ro (A.A.F.-G.); narcisa.petrescu@umft.ro (M.P.); 2 Research Center of the Institute of Cardiovascular Diseases Timisoara, 13A Gheorghe Adam Street, 300310 Timisoara, Romania; simina.crisan@umft.ro (S.C.); constantin.luca@umft.ro (C.T.L.); cristina.vacarescu@umft.ro (C.V.); dragos.cozma@umft.ro (D.C.); 3 Cardiology Clinic of the Institute of Cardiovascular Diseases Timisoara, 300310 Timisoara, Romania; 4 Department of Cardiology, “Victor Babeș” University of Medicine and Pharmacy, 300041 Timisoara, Romania; 5 CF Clinical Hospital Timișoara, 13–15 Tudor Vladimirescu Street, 300195 Timisoara, Romania; cioca.flavius@umft.ro; 6 University Discipline of Internal Medicine IV, Faculty of General Medicine, “Victor Babes” University of Medicine and Pharmacy, 2 Eftimie Murgu Square, 300041 Timisoara, Romania; lazar.daniela@umft.ro; 7 Internal Medicine Department, Clinical Emergency Military Hospital, 7 Gheorghe Lazar Street, 300080 Timisoara, Romania; 8 County Clinical Emergency Hospital of Sibiu, 550245 Sibiu, Romania; andreea.cozgarea@umft.ro; 9 Department III Functional Sciences, Medical Informatics and Biostatistics Discipline, “Victor Babes” University of Medicine and Pharmacy, 2 Eftimie Murgu Square, 300041 Timisoara, Romania

**Keywords:** HFpEF, pulse wave velocity, heart rate recovery, arterial stiffness, autonomic dysfunction, exercise testing

## Abstract

**Background/Objectives**: Heart failure with preserved ejection fraction (HFpEF) accounts for nearly half of all heart failure cases and remains challenging to diagnose and manage due to its complex, multifactorial nature. Increasing evidence highlights the significance of arterial stiffness, assessed by pulse wave velocity (PWV), and autonomic dysfunction, reflected by impaired heart rate recovery (HRR) after exercise, as relevant markers in HFpEF. This review aims to synthesize current knowledge on the diagnostic and prognostic value of PWV and HRR in HFpEF. **Methods**: A narrative literature review was conducted using PubMed to identify studies published between 2009 and 2025 that investigated PWV and HRR in patients with HFpEF or populations at risk. Included studies ranged from invasive hemodynamic measurements and cardiopulmonary exercise tests to large cohort analyses such as IDENTIFY-HF and MESA. Key findings were summarized in comparative tables. **Results**: Patients with HFpEF consistently show higher PWV than age-matched controls, supporting the concept of HFpEF as a systemic vascular disorder. Elevated PWV has been linked to increased risk of HFpEF onset and poorer outcomes. Likewise, blunted HRR indicates autonomic imbalance and is strongly associated with higher morbidity and mortality. Interventions including structured exercise training and optimized risk factor management may help improve PWV and HRR. **Conclusions**: PWV and HRR offer valuable, complementary insights for risk stratification and individualized care in HFpEF. Further research should focus on integrating these parameters into diagnostic algorithms and evaluating targeted therapies that address vascular stiffness and autonomic dysfunction.

## 1. Introduction

Heart failure with preserved ejection fraction (HFpEF) has emerged as one of the most common and challenging forms of heart failure worldwide, now accounting for nearly half of all diagnosed cases [1]. Its prevalence continues to rise [2]. Unlike heart failure with reduced ejection fraction (HFrEF), for which effective guideline-directed medical therapies have been developed, HFpEF remains notoriously difficult to diagnose and treat. Many patients experience persistent symptoms and frequent hospitalizations despite optimal management of comorbidities, underscoring the importance of a correct diagnosis in this population [3].

HFpEF is increasingly recognized as a multifactorial syndrome driven by complex interactions between cardiac and extracardiac mechanisms. Diastolic dysfunction, characterized by impaired left ventricular (LV) relaxation and increased chamber stiffness, is a central feature, leading to elevated filling pressures, left atrial enlargement, and pulmonary congestion [4]. However, contemporary research highlights that HFpEF extends beyond the myocardium. Vascular factors, such as large artery stiffening and endothelial dysfunction, contribute significantly by increasing afterload and disrupting ventricular-arterial coupling. This hemodynamic mismatch exacerbates exercise intolerance, a hallmark of the syndrome [5]. Additionally, autonomic dysregulation, particularly chronotropic incompetence and impaired parasympathetic reactivation, limits cardiac output reserve during physical activity, further reducing functional capacity [6].

Two parameters have gained increasing attention as accessible, non-invasive markers that capture key aspects of this complex pathophysiology: pulse wave velocity (PWV) and heart rate recovery (HRR). PWV is widely accepted as the gold-standard measure of arterial stiffness and has been linked to incident HFpEF, diastolic dysfunction severity, and worse prognosis [7]. Similarly, HRR is the rate at which heart rate declines after exercise and serves as a simple surrogate of cardiac autonomic balance, with slower recovery indicating vagal impairment and heightened sympathetic tone, both associated with adverse outcomes in heart failure populations [8].

HFpEF represents a heterogeneous syndrome in which ventricular, vascular, and autonomic abnormalities interact to elevate filling pressures and limit cardiac reserve. Comorbidities such as hypertension, diabetes, and obesity contribute to vascular aging and autonomic imbalance, creating the substrate on which PWV and HRR provide mechanistically relevant insight.

Despite these insights, several questions remain. How do PWV and HRR integrate with established diagnostic algorithms such as the H_2_FPEF or HFA-PEFF scores? What cut-offs are most clinically relevant in diverse patient populations? Can therapeutic strategies targeting vascular stiffness or autonomic imbalance meaningfully improve patient-centered outcomes? Addressing these gaps could refine risk stratification, guide personalized interventions, and enhance long-term management of HFpEF.

In this narrative review, we synthesize current evidence regarding the diagnostic and prognostic significance of PWV and HRR in patients with HFpEF. We also summarize key findings from recent invasive hemodynamic studies, large cohort analyses, and interventional trials, and discuss potential implications for future research and clinical practice. By integrating these physiologic markers into a broader understanding of HFpEF, we hope to contribute to a more nuanced, targeted approach to this increasingly prevalent syndrome. Although several reviews have discussed arterial stiffness or autonomic dysfunction in HFpEF, these dimensions have generally been explored in isolation. To our knowledge, no prior review has integrated pulse wave velocity (PWV), heart rate recovery (HRR), and longitudinal myocardial function within a unified pathophysiological framework—the vascular–autonomic–myocardial (VAM) triangle. This model illustrates how vascular stiffening, autonomic imbalance, and subendocardial longitudinal fiber impairment interact in a bidirectional manner to drive HFpEF progression. This integrative perspective extends beyond existing reviews by emphasizing mechanistic interconnections that may refine diagnostic assessment and phenotype-aligned therapeutic decisions.

### 1.1. Pathophysiology of HFpEF

**Figure 1:**
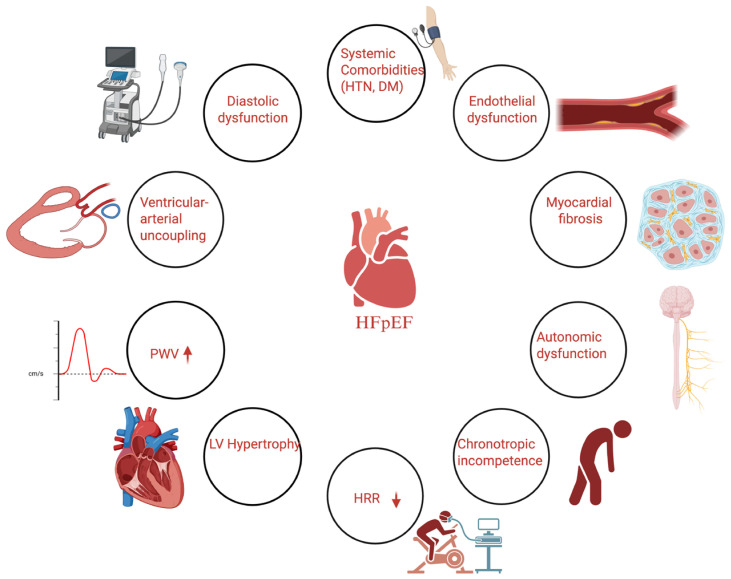
Conceptual diagram illustrating the pathophysiological mechanisms of heart failure with preserved ejection fraction (HFpEF), highlighting the role of arterial stiffness (pulse wave velocity, PWV) and autonomic dysfunction (heart rate recovery, HRR).

Heart failure with preserved ejection fraction (HFpEF) represents a multifaceted clinical syndrome that cannot be reduced to a single pathophysiological mechanism. Figure 1 illustrates the complex interplay of diastolic dysfunction, systemic comorbidities, vascular inflammation, increased arterial stiffness (pulse wave velocity), and impaired autonomic function (reflected by reduced heart rate recovery) in the pathophysiology of HFpEF, highlighting how these interrelated mechanisms contribute to exercise intolerance, adverse prognosis, and represent potential therapeutic targets.

Heart failure’s defining feature, diastolic dysfunction, reflects impaired active relaxation and increased passive stiffness of the left ventricle (LV), which together elevate LV filling pressures and promote left atrial remodeling and pulmonary venous congestion [9]. Chronic pressure overload, typically driven by long-standing hypertension, fosters concentric left ventricular hypertrophy (LVH) that further impairs compliance [10]. Importantly, modern imaging studies have revealed that a substantial subset of patients with HFpEF also exhibit subtle forms of systolic dysfunction despite preserved ejection fraction, including reduced myocardial longitudinal strain and a blunted contractile reserve during exercise [11].

Chronotropic incompetence, defined as an inadequate heart rate response to increased metabolic demand, is another hallmark of HFpEF, contributing significantly to exercise intolerance. This impaired chronotropic response is often exacerbated by the widespread use of beta-blockers in patients with coexisting hypertension or atrial fibrillation, though it may also reflect intrinsic sinus node dysfunction and abnormal autonomic modulation [12].

Beyond the myocardium, a growing body of evidence supports the concept that HFpEF is fundamentally a systemic disorder. Comorbidities such as obesity, type 2 diabetes, chronic kidney disease, and metabolic syndrome create a chronic pro-inflammatory and pro-fibrotic environment that adversely affects the vasculature [13]. Endothelial dysfunction, a common feature in HFpEF, leads to diminished nitric oxide bioavailability and increased oxidative stress, triggering vascular remodeling and stiffening. Large artery stiffening, particularly involving the aorta, increases LV afterload and disturbs ventricular-arterial coupling, worsening diastolic pressures at rest and during exertion [14]. Invasive hemodynamic studies have shown that this abnormal arterial load becomes more pronounced with exercise, when impaired vasodilation and increased wave reflections further elevate filling pressures [15].

At the microvascular level, coronary endothelial inflammation promotes diffuse myocardial fibrosis and interstitial remodeling, which can aggravate diastolic dysfunction. This “inflammatory microvascular dysfunction” model helps distinguish HFpEF from HFrEF, where primary myocyte loss predominates [16]. Peripheral factors, including reduced skeletal muscle capillary density, impaired mitochondrial function, and sarcopenia, also limit oxygen extraction, amplifying exertional intolerance [16]. This “inflammatory microvascular dysfunction” paradigm distinguishes HFpEF from the myocyte-centered pathogenesis seen in HFrEF. Additionally, impaired peripheral oxygen extraction and altered skeletal muscle metabolism further limit exercise capacity. Altogether, HFpEF emerges as a syndrome characterized by the interplay of diastolic dysfunction, arterial stiffening, and autonomic imbalance, all underpinned by a systemic inflammatory state [17].

The pathophysiology of HFpEF reflects the convergence of myocardial stiffness, impaired ventricular–arterial coupling, microvascular dysfunction, and autonomic dysregulation. These features collectively promote elevated filling pressures and exertional intolerance. PWV and HRR capture complementary aspects of this physiology—vascular stiffness and chronotropic/autonomic reserve—which are central to HFpEF progression.

### 1.2. Pulse Wave Velocity and Arterial Stiffness in Cardiovascular Assessment

Pulse wave velocity (PWV) is a well-established measure of arterial stiffness and is widely regarded as the gold-standard parameter for assessing the mechanical properties of the arterial wall. It quantifies the speed at which the pressure pulse travels through the vasculature, typically measured between two arterial sites—for example, carotid-femoral PWV (cfPWV) is the standard for evaluating central aortic stiffness [18]. A higher PWV indicates stiffer arteries, which increase systolic afterload and pulse pressure and contribute to adverse cardiac remodeling. Numerous studies have demonstrated that PWV is an independent predictor of cardiovascular events and target-organ damage [19,20,21].

**Figure 2:**
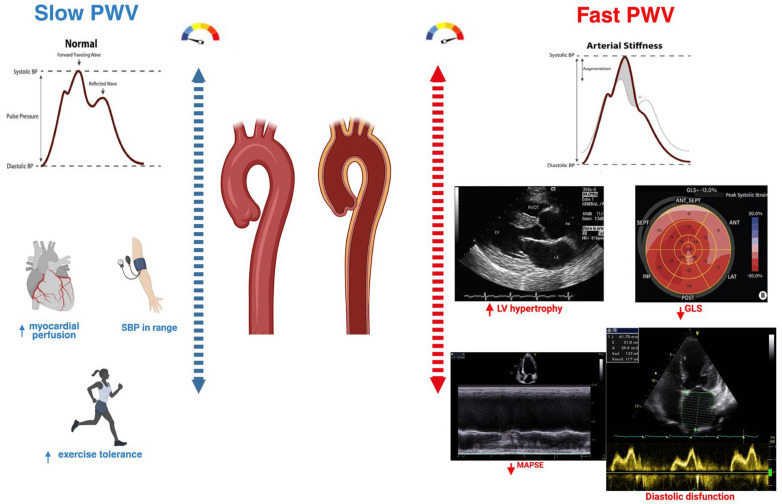
The physiological consequences of increased arterial stiffness. Comparison of slow versus fast pulse wave velocity (PWV). Slow PWV reflects normal arterial compliance, with late diastolic wave reflection supporting coronary perfusion, optimal systolic blood pressure, and preserved exercise tolerance. Fast PWV indicates increased arterial stiffness, with early systolic wave reflection leading to higher LV afterload, LV hypertrophy, impaired GLS, reduced MAPSE, and diastolic dysfunction, illustrating the mechanistic link between arterial stiffness and HFpEF.

In the setting of HFpEF, elevated aortic PWV is strongly correlated with diastolic dysfunction and plays a pathophysiological role in the development of the syndrome. Mechanistically, arterial stiffening augments systolic blood pressure and pulse pressure, increasing LV workload while reducing diastolic perfusion pressure, factors that accelerate left ventricular hypertrophy, subendocardial ischemia, and fibrotic remodeling [22] (Figure 2).

These hemodynamic consequences can lead to persistently elevated LV filling pressures both at rest and during exercise, worsening symptoms such as exertional dyspnea. Clinically, PWV is also seen as a marker of “vascular aging,” integrating the cumulative impact of age, hypertension, metabolic risk factors, and chronic inflammation on the arterial tree [2].

Although it is increasingly used in research and selected risk stratification schemes, routine clinical implementation in HFpEF is limited by heterogeneity in measurement techniques (e.g., cfPWV vs. baPWV), lack of standard thresholds specific to this population, and uncertainty about how best to modify stiffness therapeutically. Recent data suggest that interventions such as blood pressure control, RAAS blockade, exercise training, and possibly SGLT2 inhibitors may improve arterial compliance over time, but further studies are needed to confirm whether targeting PWV directly translates into meaningful improvements in clinical outcomes for patients with HFpEF.

### 1.3. Heart Rate Recovery and Exercise Testing in Cardiovascular Assessment

Heart rate recovery (HRR) refers to the rate of decline in heart rate following cessation of exercise, typically measured at one minute (HRR_1_) or two minutes (HRR_2_) post-exercise [23]. Physiologically, HRR is a reflection of parasympathetic reactivation after withdrawal of the sympathetic stimulation that predominates during exertion [24]. A faster HRR, indicated by a larger drop in heart rate immediately post-exercise, reflects a well-preserved autonomic balance, whereas a blunted HRR is a sign of impaired vagal tone and sustained sympathetic activity [8]. Impaired HRR has gained recognition as a simple yet robust prognostic tool in cardiovascular medicine.

Multiple studies have shown that an abnormal HRR is associated with increased risk of mortality and morbidity in both apparently healthy individuals and those with established heart disease [25]. In patients with heart failure, including HFpEF, HRR serves as a practical indicator of cardiac dysautonomia and offers prognostic information beyond conventional functional measures. For example, in some studies, HRR following a 6 min walk test outperformed traditional metrics like walk distance in predicting survival, suggesting its added value in risk stratification [26].

Importantly, HRR is simple to measure, requiring only a stopwatch and a reliable heart rate monitor, making it feasible even in low-resource settings. Despite its practicality, certain confounders, such as the use of beta-blockers or other rate-limiting medications, can influence HRR and should be considered in interpretation. In practice, an abnormal HRR_1_ is often defined as a decline of ≤12 beats per minute, though optimal thresholds may vary based on age, fitness level, and comorbidities [27,28].

From a mechanistic standpoint, impaired HRR in HFpEF likely reflects the interaction of elevated LV filling pressures, blunted baroreflex sensitivity, and endothelial dysfunction, all hallmarks of the syndrome’s integrated pathophysiology. This explains why a poor HRR is often accompanied by chronotropic incompetence and exercise intolerance. When used alongside other exercise-derived metrics, such as peak VO_2_ and ventilatory efficiency, HRR can offer a valuable, non-invasive window into both autonomic and hemodynamic reserve. As research progresses, incorporating HRR into standardized diagnostic and prognostic algorithms may help clinicians better identify high-risk HFpEF patients and tailor individualized therapeutic strategies.

## 2. Materials and Methods

This narrative review synthesizes current evidence on heart failure with preserved ejection fraction (HFpEF), with particular emphasis on two key physiological markers: pulse wave velocity (PWV), an established index of arterial stiffness, and heart rate recovery (HRR), an indicator of autonomic function and cardiovascular reserve.

A narrative review was performed using PubMed (2009–2025). Search terms included the following: ‘HFpEF AND pulse wave velocity’, ‘HFpEF AND arterial stiffness’, ‘HFpEF AND heart rate recovery’, ‘chronotropic incompetence AND HFpEF’, and MeSH combinations (‘Heart Failure/physiopathology’ AND ‘Pulse Wave Analysis’). Only PubMed was searched; Embase, Web of Science, and Cochrane Library were not examined, which represents a methodological limitation.

Selection focused on clinically relevant studies, including large observational cohorts (such as MESA and PARAGON-HF), randomized controlled trials, invasive hemodynamic assessments, and cardiopulmonary or submaximal exercise testing that examined the diagnostic, prognostic, or therapeutic implications of PWV and HRR in patients with HFpEF or at-risk populations. Titles and abstracts were reviewed independently by the authors, and any disagreements were resolved by consensus. Gray literature, non-peer-reviewed reports, and unpublished data were excluded. Although a formal systematic process such as PRISMA was not applied, the search strategy aimed to capture a representative and balanced overview of the topic to highlight clinically meaningful findings and identify areas where further research is needed.

## 3. Results

### 3.1. HFpEF and Pulse Wave Velocity: Insights from Recent Studies

Pulse wave velocity represents an important tool for quantifying arterial stiffness, which is considered a key pathophysiological pillar in the development of heart failure [29]. Arterial stiffness can be measured by several non-invasive methods, one of the most common being carotid-femoral pulse wave velocity (cf-PWV), which reflects thoracic and abdominal aortic stiffness and serves as a predictor of cardiovascular events [30]. Brachial-ankle pulse wave velocity (ba-PWV) is another method used to assess arterial stiffness, more commonly applied in Asia. Unlike cf-PWV, this technique measures a longer arterial pathway and provides a better reflection of arterial stiffness, thereby serving as an indicator of both central and peripheral arterial stiffness [31].

In recent years, equations incorporating chronological age and blood pressure have been developed to estimate pulse wave velocity (ePWV). In this context, Heffernan et al. employed ePWV to demonstrate that individuals with elevated ePWV exhibit a higher incidence of HFpEF and have a fourfold increased risk of developing HFpEF [32].

Cut-off values for ba-PWV have been proposed, with <14 m/s considered normal and >18 m/s considered abnormal. However, further studies are needed before these thresholds can be implemented [31,33]. Tanaka et al. also proposed a cut-off value for cf-PWV of >10 m/s as abnormal and associated with increased cardiovascular risk [33].

The 2024 European Society of Cardiology Guidelines on Elevated Blood Pressure and Hypertension consider ba-PWV values > 14 m/s and cf-PWV values > 10 m/s as criteria that may be used to define hypertension-mediated organ damage.

Arterial stiffness plays a particularly salient role in the pathophysiology of HFpEF. Multiple studies have shown that patients with HFpEF tend to have higher PWV, indicating stiffer arteries, compared to age-matched individuals without heart failure [14,32,34].

This is largely attributed to the high burden of comorbidities such as hypertension and diabetes, which accelerate vascular aging and endothelial dysfunction. For instance, a recent study (IDENTIFY-HF) reported that arterial stiffness progressively increases as vascular comorbidities accumulate, with HFpEF patients exhibiting the highest levels of arterial stiffness, whereas patients with HFrEF showed near-normal levels. This finding supports the concept of HFpEF as a “disease of the vasculature” driven by comorbidity-related vascular remodeling [35]. Direct comparisons further reinforce this point. Desai et al. found that patients with hypertensive HFpEF had significantly greater central aortic stiffness compared to healthy normotensive controls [34].

Table 1 summarizes key studies evaluating arterial stiffness in HFpEF.

Comorbidities such as aging, hypertension, and diabetes promote arterial stiffening, which raises systolic load and widens pulse pressure. The resulting left ventricular hypertrophy and diastolic dysfunction, along with reduced coronary perfusion during diastole, contribute directly to the pathogenesis of HFpEF. Endothelial inflammation and oxidative stress act as important mediators of this process [5,18].

A landmark invasive study by Reddy et al. used pressure catheters to assess arterial function in HFpEF. In 98 HFpEF patients compared to 22 hypertensive controls, arterial compliance was significantly reduced in the HFpEF group and strongly correlated with higher LV filling pressures. Notably, during exercise, HFpEF patients exhibited a further loss of arterial compliance, reflecting worsening stiffness, whereas hypertensive controls maintained stable hemodynamics [14].

In a related French study, exercise induced a 20% increase in aortic PWV in HFpEF patients, while control subjects showed a slight decrease, likely due to appropriate vasodilation. These findings highlight a key pathophysiologic feature: patients with HFpEF are unable to vasodilate properly under stress, which exacerbates afterload and contributes to pulmonary congestion during exertion. Consistent with these hemodynamic studies, measures of arterial stiffness have demonstrated clear prognostic significance in HFpEF [37].

Moreover, Tokitsu et al. followed 502 HFpEF patients for approximately three years and found a higher rate of cardiovascular events at the extremes of arterial stiffness, specifically, when baPWV was very low (<1300 cm/s) or very high (>1900 cm/s). This U-shaped relationship suggests that both severe stiffening and unusually low PWV (possibly reflecting poor cardiac output) portend worse outcomes, whereas moderate arterial compliance appears optimal [21].

Pulmonary hypertension is a common condition in patients with HFpEF, increasing the risk of cardiovascular mortality. Nakamura et al. performed right heart catheterization and measured pulse wave velocity using baPWV in 198 patients with HFpEF, demonstrating that patients with increased systemic arterial stiffness develop precapillary pulmonary hypertension [36].

Moreover, pulse wave velocity measured as ePWV was associated with the onset of heart failure, making this parameter useful not only for monitoring patients with heart failure but also for assessing the risk of developing the condition [7].

Finally, large-scale epidemiologic data from the Multi-Ethnic Study of Atherosclerosis (MESA) support these findings: individuals in the highest quartile of estimated PWV had nearly fourfold higher risk of developing HFpEF over 12 years compared to those in the lowest quartile. Taken together, these insights reinforce that arterial stiffness is intimately linked with HFpEF—serving both as a contributing factor to disease pathogenesis and as a marker of prognosis [38].

### 3.2. HFpEF and Heart Rate Recovery: Insights from Recent Studies

The study conducted by Cole et al. was the first to introduce the concept of heart rate recovery, using a cut-off value of 12 beats per minute, and demonstrated that a reduced value of this parameter was a strong predictor of cardiovascular mortality [27].

Over time, multiple studies have investigated heart rate recovery in patients with heart failure and have demonstrated that a reduced value of this index is associated with increased cardiovascular mortality [39,40,41]. However, the majority of these studies have been conducted on cohorts of patients with heart failure with reduced ejection fraction, and further research is needed to evaluate this parameter in patients with preserved ejection fraction.

Exercise intolerance remains one of the most prominent clinical features of HFpEF, and impaired chronotropic and autonomic responses play a central role in this limitation [42]. Multiple studies have confirmed that patients with HFpEF frequently exhibit abnormal heart rate recovery (HRR) after exercise, which reflects their blunted autonomic reflexes [6,26,43,44].

Phan et al., for example, performed maximal cardiopulmonary exercise testing in HFpEF patients not receiving rate-limiting medications and matched controls. They found that chronotropic incompetence was present in the majority of HFpEF patients, who reached a significantly lower percentage of their age-predicted maximal heart rate during exercise compared to controls. Not surprisingly, peak VO_2_ was approximately 35% lower in the HFpEF group, underscoring reduced aerobic capacity. Importantly, HRR at one minute (HRR_1_) was abnormal in 23% of HFpEF patients versus only 2% of controls (*p* = 0.01). On average, HFpEF patients demonstrated a smaller drop in heart rate after exercise, consistent with delayed vagal reactivation. These findings provided early direct evidence of intrinsic autonomic dysfunction in HFpEF, independent of medication effects, and similar to patterns seen in hypertensive heart disease [43]. Subsequent studies have reinforced HRR as a robust risk marker in HFpEF, comparable to its established role in HFrEF.

In a 2013 study, 258 chronic heart failure patients (including 42 with HFpEF) underwent both a 6 min walk test and treadmill cardiopulmonary exercise testing. HRR after exercise emerged as the strongest predictor of adverse events on both tests, outperforming traditional metrics such as 6MWT distance or peak VO_2_ [26]. On multivariate analysis, a blunted HRR, defined by an established cutoff, was associated with markedly higher rates of mortality and hospitalization, independent of ejection fraction. The authors even concluded that HRR should replace 6MWT distance as the reference criterion for risk stratification in heart failure. Although that study pooled HFpEF and HFrEF patients, the inclusion of the HFpEF subgroup suggests the prognostic relevance of HRR applies across the entire spectrum of heart failure phenotypes. Additional analyses have linked higher E/E′ ratios, an echocardiographic marker of elevated diastolic filling pressures, with blunted HRR, reinforcing the physiologic connection between diastolic dysfunction and impaired autonomic recovery [26,44]. These relationships make physiologic sense: elevated filling pressures and baroreceptor-mediated reflex sympathetic activation may dampen parasympathetic reactivation after exercise, resulting in a slower HRR. This integrated view highlights why HRR can provide unique prognostic information alongside other exercise-derived metrics [44].

The most common assessments of HRR involve its measurement at 1 min (HRR1) or 2 min (HRR2) following the cessation of exercise. The majority of studies in the literature have evaluated this parameter in patients with heart failure with reduced ejection fraction, whereas relatively few investigations have examined it in patients with HFpEF.

For this purpose, a novel parameter was proposed [45], termed the heart rate recovery index (HRRI). It is derived by dividing the ascending slope by the descending slope during the exercise test. This parameter was evaluated in patients with heart failure undergoing cardiac resynchronization therapy, where it was demonstrated that the index was significantly higher among responders compared to non-responders [45].

Patients with heart failure with preserved ejection fraction are typically characterized by the presence of multiple comorbidities, which often necessitate the use of beta-blocker therapy. Given the frequent administration of these medications in this patient population, the question arises as to whether beta-blockers may influence heart rate recovery (HRR) and the chronotropic incompetence observed in these patients, thereby potentially exacerbating exercise intolerance.

In this context, heart rate recovery (HRR) was analyzed in 174 patients with heart failure with preserved ejection fraction (HFpEF), of whom 59 were receiving beta-blocker therapy and 115 were not. Although resting heart rate was lower among patients treated with beta-blockers, HRR did not appear to be influenced by beta-blocker therapy between the two groups [46].

On the other hand, Palau et al. analyzed a cohort of patients with heart failure with preserved ejection fraction in whom beta-blocker therapy was withdrawn, demonstrating that in certain categories of patients with chronotropic incompetence, discontinuation of treatment led to a short-term improvement in functional capacity [28].

However, further studies evaluating heart rate recovery in patients with HFpEF are warranted to determine whether beta-blockers may influence this parameter.

Table 2 summarizes key studies evaluating HRR in HFpEF and mixed HF cohorts. Looking forward, incorporating HRR into multi-parameter diagnostic and risk prediction algorithms could enhance the accuracy of HFpEF assessment and help guide more tailored therapeutic interventions.

## 4. The Vascular–Autonomic–Myocardial Triangle

The subendocardial fibers represent the innermost layer of myocardial fibers, located adjacent to the endocardium. In the left ventricle, these fibers are arranged in a right-handed helical orientation, an organization that plays a crucial role in facilitating longitudinal shortening and promoting myocardial contraction from the base toward the apex. This specific arrangement ensures an efficient and coordinated contraction of the left ventricle [47,48].

The longitudinal fibers are highly sensitive to ischemia, particularly in conditions of pressure overload or increased wall stress, due to their elevated metabolic demand and relatively poor vascular supply. The earliest signs of impairment in these fibers are reflected by alterations in longitudinal deformation, as demonstrated echocardiographically through parameters such as global longitudinal strain (GLS) or mitral annular plane systolic excursion (MAPSE), as well as the onset of diastolic dysfunction, one of the hallmark features of heart failure with preserved ejection fraction (HFpEF) [49].

Heart failure with preserved ejection fraction is increasingly recognized as a multifactorial, systemic disease resulting from the complex interplay of several pathophysiological mechanisms, among which autonomic, myocardial, and vascular dysfunctions play the most significant roles.

Pulse wave velocity (PWV) represents the direct method for assessing arterial stiffness, with increased values indicating elevated aortic and central arterial rigidity. Pathophysiologically, increased aortic stiffness leads to elevated left ventricular afterload and augmented wall stress, which in turn contribute to coronary microcirculatory disturbances and subsequent impairment of myocardial longitudinal fibers, ultimately exacerbating heart failure progression [50].

A 2025 study by Wang et al. analyzed ventriculo–arterial coupling using the ePWV/GLS ratio in 131 patients with heart failure encompassing the entire spectrum of ejection fraction, compared to a control group. A higher ePWV/GLS ratio was associated with heart failure progression, with patients exhibiting reduced ejection fraction demonstrating the highest ePWV/GLS values. Furthermore, this ratio showed significant correlations with other biological markers (NT-proBNP) and echocardiographic parameters (E/e′) [51].

Heart rate recovery is a parameter used to assess autonomic dysfunction and is frequently reduced in patients with heart failure with preserved ejection fraction. A diminished heart rate recovery is associated with increased sympathetic tone, which contributes to delayed left ventricular relaxation and elevated left ventricular filling pressures [52].

Several studies have demonstrated that diastolic dysfunction is associated with an abrupt reduction in heart rate recovery, showing a strong correlation with echocardiographic indices such as E/e′ and E/A, which are established markers of elevated left ventricular filling pressures [53,54].

Thus, a reduced heart rate recovery reflects increased sympathetic tone, which is associated with microvascular vasoconstriction and shortened ventricular filling time, ultimately leading to impairment of longitudinal myocardial fibers and further exacerbation of heart failure.

PWV may identify patients with advanced vascular aging or hypertension-mediated organ damage, informing intensification of BP control or RAAS/SGLT2 inhibitor initiation. HRR provides insight into chronotropic reserve, supporting decisions on beta-blocker deprescribing and exercise prescription. Both markers may complement H_2_FPEF and HFA-PEFF by refining vascular and autonomic phenotyping, particularly in borderline diagnostic cases.

We propose a pathophysiological construct termed the vascular–autonomic–myocardial (VAM) triangle, which delineates the complex interrelationships among arterial stiffness (PWV), autonomic dysfunction as reflected by heart rate recovery (HRR), and subendocardial myocardial fiber impairment (GLS, MAPSE). Each component exerts bidirectional influences on the others, collectively fostering a self-perpetuating cycle that contributes to the development and progression of heart failure with preserved ejection fraction (HFpEF). PWV aligns with structural/functional domains of HFA-PEFF and the extracardiac burden reflected in H_2_FPEF. HRR captures chronotropic reserve, a determinant of exertional intolerance not explicitly included in these scores. Incorporating PWV and HRR as adjunctive markers may refine phenotyping, especially in borderline or discordant cases. PWV varies across cfPWV, baPWV, and estimated PWV, with device-specific thresholds limiting comparability. HRR differs depending on maximal vs. submaximal testing, timing of recovery (1 vs. 2 min), and beta-blocker influence. These methodological inconsistencies underscore the need for standardized protocols.

**Figure 3:**
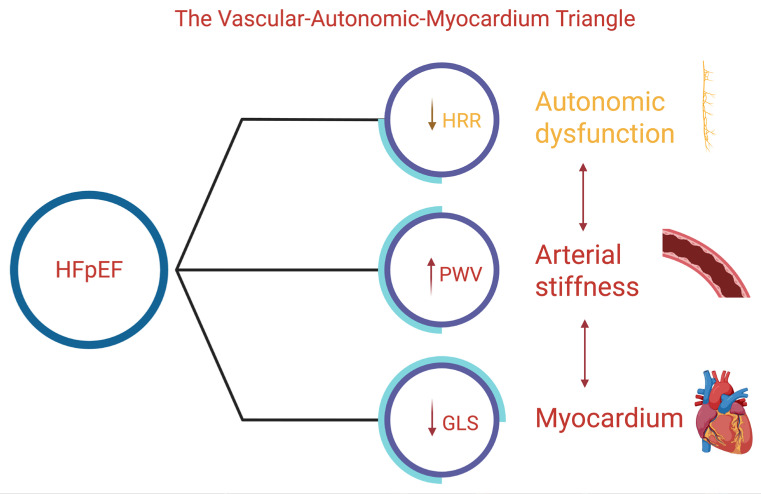
Schematic representation of the vascular–autonomic–myocardial triangle. Decreased heart rate recovery (HRR) reflects autonomic dysfunction; increased pulse wave velocity (PWV) represents arterial stiffness; and reduced global longitudinal strain (GLS) indicates myocardial impairment. The arrows denote the direction of dysfunction in each component, illustrating their interconnected contribution to the HFpEF pathophysiology.

The VAM triangle extends existing HFpEF frameworks by integrating vascular stiffness, autonomic reactivation, and longitudinal myocardial function into a unified construct. Unlike the microvascular inflammation paradigm or ventricular–arterial coupling models—which focus primarily on endothelial injury or hemodynamic load—the VAM model highlights reciprocal interactions across vascular, autonomic, and myocardial domains, offering a more comprehensive representation of HFpEF pathophysiology (Figure 3).

## 5. Discussion

This narrative review highlights how recent evidence strengthens the concept that arterial stiffness and autonomic imbalance play key roles in the multifactorial pathophysiology of heart failure with preserved ejection fraction (HFpEF). The consistent finding that pulse wave velocity (PWV) is significantly higher in HFpEF patients compared to matched controls supports earlier hypotheses that HFpEF is not merely a myocardial disorder but rather a systemic syndrome characterized by vascular aging and impaired ventricular–arterial coupling [18,19]. These results align with prior hemodynamic studies, such as those by Reddy et al. and others, which demonstrated reduced arterial compliance at rest and a further decline during exercise [14].

This evidence reinforces the idea that stiffened large arteries exacerbate diastolic dysfunction and limit cardiac reserve under stress, contributing directly to exercise intolerance.

Similarly, our synthesis confirms that abnormal heart rate recovery (HRR) after exercise reflects an intrinsic autonomic dysfunction in HFpEF, echoing early findings by Phan et al. [43] and Cahalin et al. [44]. Recent analyses have shown that HRR offers incremental prognostic value beyond traditional exercise measures such as peak VO_2_ or six-minute walk distance, suggesting that HRR may better capture the integrated autonomic and hemodynamic limitations that characterize HFpEF [26].

In contrast to prior reviews that examined PWV or HRR separately, our synthesis introduces the VAM triangle as an integrative construct linking large-artery stiffness, autonomic reactivation dynamics, and subendocardial longitudinal dysfunction. Existing models—such as microvascular inflammation or ventricular–arterial coupling frameworks—do not explicitly incorporate these reciprocal interactions. The VAM framework therefore provides a broader mechanistic lens through which HFpEF pathophysiology can be interpreted.

In the broader clinical context, these findings support the rationale for incorporating simple, non-invasive measures such as PWV and HRR into diagnostic and risk stratification models, complementing established tools like echocardiography and natriuretic peptide levels. However, these markers are not yet routinely used in practice. There remains a need for standardized measurement protocols, population-specific thresholds, and consensus on how to integrate them into multimodal scores like H_2_FPEF or HFA-PEFF.

Future research should clarify whether interventions that improve vascular compliance or autonomic function—for example, structured exercise training, aggressive risk factor control, or novel therapies targeting endothelial dysfunction—can translate into meaningful improvements in morbidity and mortality for HFpEF patients. Well-designed randomized trials are needed to validate PWV and HRR not only as markers of disease severity but also as potential therapeutic targets. Given the heterogeneity of HFpEF phenotypes, exploring how these markers might identify subgroups who would benefit most from specific strategies should be a priority. Exercise trials vary widely in protocols and seldom examine PWV or HRR as primary endpoints. RAAS blockade yields inconsistent effects on arterial stiffness, and benefits depend largely on BP reduction. SGLT2 inhibitors improve hemodynamics and functional capacity, but PWV/HRR evidence remains observational. Interventional trials directly testing whether modifying these markers improves outcomes are needed.

Taken together, our review suggests that evaluating PWV and HRR alongside conventional assessments could help clinicians better understand the complex pathophysiology of HFpEF and guide more personalized, physiology-based management of this challenging syndrome.

### Limitations

PWV and HRR may inform vascular and autonomic phenotyping, refine risk stratification, and guide therapy tailoring in HFpEF. Research gaps include lack of standardized measurement protocols, absence of validated HFpEF-specific cutoffs, limited longitudinal data, and the need for interventional trials testing whether modifying PWV or HRR improves outcomes. This narrative review is subject to selection and reporting bias. Only PubMed was searched, which may have led to omission of relevant studies. HFpEF definitions, PWV methodologies, and HRR protocols were heterogeneous across studies, limiting comparability.

Evidence specifically evaluating HRR in HFpEF remains sparse, restricting phenotype-specific conclusions.

## 6. Conclusions

Heart failure with preserved ejection fraction remains a complex and heterogeneous syndrome at the intersection of myocardial, vascular, and autonomic dysfunction. Current evidence suggests that pulse wave velocity and heart rate recovery provide valuable, complementary insights into these processes, with clear diagnostic and prognostic implications. Incorporating these simple, non-invasive measures into clinical practice may help refine patient profiling and risk stratification. Future studies should aim to establish clear cut-offs, validate practical algorithms, and determine whether targeting arterial stiffness and autonomic imbalance can translate into improved outcomes for patients living with HFpEF.

## Figures and Tables

**Table 1:** Key Studies on Arterial Stiffness (PWV) in HFpEF.

Study Title	Authors	Year	Population	Key Findings on PWV
Estimated pulse wave velocity predicts mortality in patients with heart failure with preserved ejection fraction	Xue et al. [20]	2024	1764 patients with HFpEF	In patients with HFpEF, elevated ePWV predicted both all-cause and cardiac mortality
Estimated pulse wave velocity and incident heart failure and its subtypes: Findings from the multi-ethnic study of atherosclerosis	Heffernan et al. [32]	2023	6814 community adults (MESA study; 138 developed HFpEF)	Over 12.5 years, individuals with higher baseline PWV had a greater incidence of HFpEF. The highest quartile of PWV had approximately a fourfold higher risk of developing HFpEF versus the lowest quartile, implicating arterial stiffness as a risk factor for HFpEF onset.
Pulmonary vascular resistance is associated with brachial-ankle pulse-wave velocity and adverse clinical outcomes in patients with heart failure with preserved ejection fraction	Nakamura et al. [36]	2019	198 HFpEF patients	Systemic arterial stiffness has been associated with increased pulmonary vascular resistance and with the development of precapillary pulmonary hypertension in patients with HFpEF.
Clinical significance of brachial-ankle pulse wave velocity in patients with heart failure with preserved left ventricular ejection fraction	Tokitsu et al. [21]	2018	502 HFpEF patients (3-year follow-up)	Prognostic J-curve for stiffness: Both very high and very low baPWV were associated with increased cardiovascular events in HFpEF. Intermediate stiffness conferred the lowest risk, suggesting arterial stiffness assessment can help stratify HFpEF prognosis.
Arterial stiffening with exercise in patients with heart failure and preserved ejection fraction	Reddy et al. [14]	2017	98 HFpEF vs. 22 hypertensive controls	Arterial compliance was lower in HFpEF than in hypertensive controls, independent of blood pressure. On exercise, HFpEF patients showed an increase in aortic stiffening, whereas controls had a slight decrease, leading to worse ventricular-arterial coupling in HFpEF.
Central aortic stiffness is increased in patients with heart failure and preserved ejection fraction	Desai et al. [34]	2009	53 patients, of whom 16 with HFpEF and HTN, 23 with HTN but without HFpEF, and 14 had healthy controls	HFpEF patients had significantly increased central aortic stiffness compared to healthy age-matched controls (higher carotid-femoral PWV), indicating pronounced arterial stiffening in HFpEF.

PWV—pulse wave velocity; HFpEF—heart failure with preserved ejection fraction; HTN—arterial hypertension; ePWV—estimated pulse wave velocity; baPWV—brachial-ankle pulse wave velocity; MESA—Multi-Ethnic Study of Atherosclerosis.

**Table 2:** Key Studies on Heart Rate Recovery (HRR) in HFpEF.

Study Title	Authors	Year	Population	Key Findings on HRR
Role of heart rate recovery in chronic heart failure: Results from the MyoVasc study	Velmeden et al. [6]	2025	1289 patients with HF across the entire spectrum of ejection fraction	HRR60 demonstrated a strong association with worsening heart failure, particularly in patients with HFpEF
Impact of beta-blockers on heart rate and oxygen uptake during exercise and recovery in older patients with heart failure with preserved ejection fraction	Maldonado-Martin et al. [46]	2020	174 HFpEF patients (BB = 59; NBB = 115)	Treatment with beta-blockers does not affect HRR in patients with HFpEF compared to those not receiving beta-blocker therapy.
Predictors of abnormal heart rate recovery in patients with heart failure, reduced and preserved ejection fraction	Cahalin et al. [44]	2014	240 HF patients (200 HFrEF, 40 HFpEF)	Correlates of abnormal HRR: In this cohort, worse HRR was associated with poorer exercise performance and echocardiographic indices. Notably, a high E/E′ (reflecting diastolic dysfunction) and the presence of exercise oscillatory ventilation were independent predictors of abnormal HRR, linking impaired HRR with HF severity in both HFpEF and HFrEF.
Heart rate recovery after the 6 min walk test, rather than distance ambulated, is a powerful prognostic indicator in heart failure with reduced and preserved ejection fraction: a comparison with cardiopulmonary exercise testing	Cahalin et al. [26]	2013	258 HF patients (216 HFrEF, 42 HFpEF)	HRR as top prognostic marker: HRR_1_ after both 6MWT and CPX was the strongest predictor of mortality or hospitalization in multivariate models (*p* < 0.001). A blunted HRR conferred significantly higher risk, overshadowing 6MWT distance and peak VO_2_. This suggests HRR is a powerful integrative measure of risk in HF, including HFpEF.
Impaired heart rate recovery and chronotropic incompetence in patients with heart failure with preserved ejection fraction	Phan et al. [43]	2010	41 HFpEF vs. 41 controls (no β-blockers)	Chronotropic incompetence and slow HRR in HFpEF: HFpEF patients used a significantly smaller fraction of heart rate reserve during exercise. Abnormal 1 min HRR (≤12 bpm drop) was observed in 23% of HFpEF patients vs. 2% of controls, indicating impaired vagal rebound in HFpEF.

HRR—heart rate recovery; HRR60—heart rate recovery at 60 s after cessation of physical exertion; HFpEF—heart failure with preserved ejection fraction; HRR_1_—heart rate recovery after cessation of physical effort; 6MWT—6 min walk test; CPX—cardiopulmonary exercise testing; VO_2_—oxygen uptake; HF—heart failure; HFrEF—heart failure with reduced ejection fraction; BB—beta-blockers; NBB—no beta-blockers.

## Data Availability

No new data were created or analyzed in this study. Data sharing is not applicable to this article.

## Abbreviations

The following abbreviations are used in this manuscript:

HFpEF	Heart Failure with Preserved Ejection Fraction
HFrEF	Heart Failure with Reduced Ejection Fraction
PWV	Pulse Wave Velocity
HRR	Heart Rate Recovery
LV	Left Ventricle/Left Ventricular
LVH	Left Ventricular Hypertrophy
baPWV	Brachial-Ankle Pulse Wave Velocity
cfPWV	Carotid-Femoral Pulse Wave Velocity
CPET	Cardiopulmonary Exercise Test
6MWT	Six-Minute Walk Test
E/E’	Ratio of mitral inflow E velocity to mitral annular E’ (diastolic function index)
RCT	Randomized Controlled Trial
MESA	Multi-Ethnic Study of Atherosclerosis
